# Supplementary material for: A Critical View of the Application of the APEX Software (Aqueous Photochemistry of Environmentally-Occurring Xenobiotics) to Predict Photoreaction Kinetics in Surface Freshwaters
Source: Molecules. 2019 Dec 18;25(1):9. doi: 10.3390/molecules25010009 (PMC7017383; doi:10.3390/molecules25010009)
Supplement: Supplementary file 1 [file molecules-25-00009-s001.zip › 3.2.4_gcc-4.4.0.zip!/3.2.4_gcc-4.4.0/3.2.4_gcc-4.4.0/doc/octave/html/interpreter/Comma-Separated-Lists-Generated-from-Structure-Arrays.html]

Comma Separated Lists Generated from Structure Arrays - Untitled


Previous: Comma Separated Lists Generated from Cell Arrays,
Up: Comma Separated Lists


---

#### 6.3.2 Comma Separated Lists Generated from Structure Arrays

Structure arrays can equally be used to create comma separated
lists. This is done by addressing one of the fields of a structure
array. For example

```
     x = ceil (randn (10, 1));
     in = struct ("call1", {x, 3, "last"},
                  "call2", {x, inf, "first"});
     out = struct ("call1", cell (2, 1), "call2", cell (2, 1));
     [out.call1] = find (in.call1);
     [out.call2] = find (in.call2);
```
